# Effective Measurement of the Influence of an Ovoidal Particle Shape on the Tortuosity and Permeability: Theoretical and Numerical Studies

**DOI:** 10.3390/ma19163498

**Published:** 2026-08-18

**Authors:** Jiangnan Hao, Xiangshang Chen, Jianjun Lin

**Affiliations:** 1 School of Materials Science and Engineering, Hebei University of Technology, Tianjin 300401, China; 18633828898@163.com; 2 School of Civil and Transportation Engineering, Hebei University of Technology, Tianjin 300401, China; 3 Key Laboratory of Green Construction and Intelligent Maintenance for Civil Engineering of Hebei Province, School of Civil Engineering & Mechanics, Yanshan University, Qinhuangdao 066004, China; linjianjun@ysu.edu.cn

**Keywords:** tortuosity, lattice Boltzmann method (LBM), permeability, Kozeny–Carman formula

## Abstract

An ovoid is a common particle, which is usually formed by constant extrusion and friction during running water handling. However, there is still debate over how the form of ovoidal particles affects the tortuosity and water permeability of particle packing systems. The tortuosity and permeability of the particle packing system are examined in relation to the shape and volume fraction of the particles in this work. The ovoid particle packing system is built using the Monte Carlo approach, and the tortuosity is obtained numerically. Then, the accuracy of the tortuosity prediction model is evaluated by comparing the theoretical derived tortuosities with the simulated results in this work and other literature. By combining the widely used Kozeny–Carman (K-C) formula with the derived theoretical tortuosity prediction model, we develop a modified K-C formula to predict the permeability of ovoidal particle packing systems. By comparing the model outputs with published experimental data and self-conducted lattice Boltzmann method (LBM) numerical simulations, we verify that the modified K-C formula achieves high prediction accuracy. According to the findings, when the aspect ratio *c*/*a* rises, the tortuosity first decreases and then increases, and the permeability first increases and then decreases.

## 1. Introduction

Particle packing systems are widely found in natural and manufactured porous composites, such as soil [1], sand [2,3], and cementitious materials [4,5]. Porous composites should, in general, be durable to lower maintenance expenses while in use. The durability of porous composite is closely related to its internal water transport behavior, since water is not only a direct participant in physical deterioration but also provides the medium for aggressive media to invade into the composites [6,7,8,9,10]. Due to the variable shape and spatial arrangement of the particles, the water transport path is winding and rugged, which makes it hard to accurately assess the porous composites’ permeability. Therefore, controlling the permeability and durability of porous composites requires precisely assessing how the particle form affects the tortuosity of water transport pathways and determining the quantitative link between tortuosity and permeability.

The definitions of water transport paths include both linear tortuosity *τ_l_* [11] and planar tortuosity *τ_p_* [12]. The linear tortuosity *τ_l_* is the proportion of the total length of the transport path *L_e_* to the length of the sample edges *L_s_*, as shown in Equation (1a); the linear tortuosity *τ_l_* has good applicability in characterizing the transport path tortuosity in two-dimensional space. In order to characterize the transport path tortuosity in the three dimensional porous composite, Stroeven [12] and Li et al. [13] adopted the planar tortuosity *τ_p_*, which was considered the proportion of total area of the fluid and gas flowing to the cross-sectional area of the sample, as shown in Equation (1b). Compared with the linear tortuosity *τ_l_*, the planar tortuosity *τ_p_* can more comprehensively characterize the porous composite transport paths with tortuosity; therefore, the planar tortuosity *τ_p_* is employed in this work.

(1a) $$\tau_{l}=\frac{L_{e}}{L_{s}}$$

(1b) $$\tau_{p}=\frac{S_{e}}{A_{s}}$$

where *τ_l_* is the linear tortuosity; *L_e_* is the total length of the tortuous path; *L_s_* is the sample length; *τ_p_* is the planar tortuosity; *S_e_* is the total area on the tortuous surface; *A_s_* is the cross-sectional area of the sample.

The theoretical derivations and numerical methods are usually adopted to investigate the tortuosity of particle packing system. For example, a model of tortuosity is presented [14], according to regular triangle arrangement and square pitch arrangement in two-dimensional microstructures. Wu et al. [15,16] studied how the representative elementary volume affects the degree of transport path tortuosity. Yu and Li [17] developed an overlapping particle packing model and obtained an approximation of the tortuosity near the percolation threshold. Assuming particles are unrestrictedly in porous composites, Yun et al. [18] propose a linear tortuosity prediction formula for spherical, cubic, and plate packing systems. It is challenging to efficiently determine the tortuosity of the actual porous composite using the aforementioned tortuosity prediction models since the present models fix the particle arrangement, which is inconsistent with an actual random particle packing system. To determine how ovoidal particle constitution affects the tortuosity and permeability, this work generates an ovoidal particle packing system based on the mathematical expression of the ovoid particles [19], as shown in Equation (2).

(2) $$F_{ovoid}(x,y,z)={\left(\frac{1}{T\frac{z}{c}+1}\times \frac{x}{a}\right)}^{2}+{\left(\frac{1}{T\frac{z}{c}+1}\times \frac{y}{b}\right)}^{2}+{\left(\frac{z}{c}\right)}^{2}-1=0$$

where *F_ovoid_* is the formula for the ovoid particle. The ovoid’s semi-axis lengths in the *x*, *y*, and *z* axes are expressed as *a*, *b*, and *c*, respectively; in addition, the semi-axis length *b* is equal to the semi-axis length *c* in this work; and *T* is the tapering parameter.

To illustrate how the tortuosity of the transport path affects the permeability, a series of empirical/semi-empirical models are proposed [20,21,22]. One of the most widely used models is the K-C formula [6], as shown in Equation (3). In the initial study, the K-C formula constant *C_k_* ≈ 5 was an accepted empirical value [23,24,25]. However, with further study, it was found that the K-C formula constant *C_k_* is not a constant but a quantity [26,27] that varies with the internal composition of the particle packing system. For example, for the peat layer, Mathavan et al. [28] discovered *C_k_* = 3.4. Kyan et al. [29] discovered that when the particle volume fraction *V_V,g_* was 0.05 or lower, there was an exponential connection between *C_k_* and *V_V,g_*. To determine the K-C formula constant *C_k_* for different particle packing systems, to measure the link between the tortuosity *τ* and the K-C formula constant *C_k_*, Equation (4) was developed [30]. Therefore, it will be possible to estimate the permeability *k* of the ovoid particle packing system by quantitatively describing the impact of the particle shape and volume fraction on the tortuosity *τ* and the shape parameter *C_f_*.

(3) $$k=\frac{{\left(1-V_{V,g}\right)}^{3}}{C_{k}V_{V,g}^{2}S_{V,g}^{2}}$$

(4) $$C_{k}=C_{f}\tau^{2}$$

where *k* is the permeability; *V_g_* is the volume fraction of the particles; *S_V,g_* is the specific surface area of the particles; *C_k_* is the K-C formula’s constant, as shown in Equation (4); *C_f_* is the shape parameter; and *τ* is the tortuosity.

It can be found that there are some limitations of traditional regular tortuosity–permeability prediction models: fixed constant K-C formula and incomplete multi-parameter coupling research. To characterize the effect of the internal composition (i.e., particle shapes, volume fractions, and size distributions) of ovoid particles on the planar tortuosity of the transport path and water permeability, this work is organized into the following sections: Section 2 theoretically derives the transport path tortuosity in the ovoid particle packing system and determines the theoretical model’s dependability through comparing the theoretical model with the numerical simulation results, which reveals the effect of the internal composition of the particle packing system on the planar tortuosity *τ_p_*. Section 3 determines the permeability *k* of the particle packing system using the lattice Boltzmann method (LBM). A modified K-C formula for the mono-sized particle packing system (MPPS) and poly-sized ovoid particle packing system (PPPS) is proposed by combining the theoretical tortuosity prediction model with the shape parameter *C_f_* prediction formula. The relationship between *C_f_* and the shape and volume fraction of the ovoid particles is determined.

## 2. A Theoretical Model of Tortuosity

### 2.1. Mono-Sized Ovoid Particle Packing System (MPPS)

**Figure 1:**
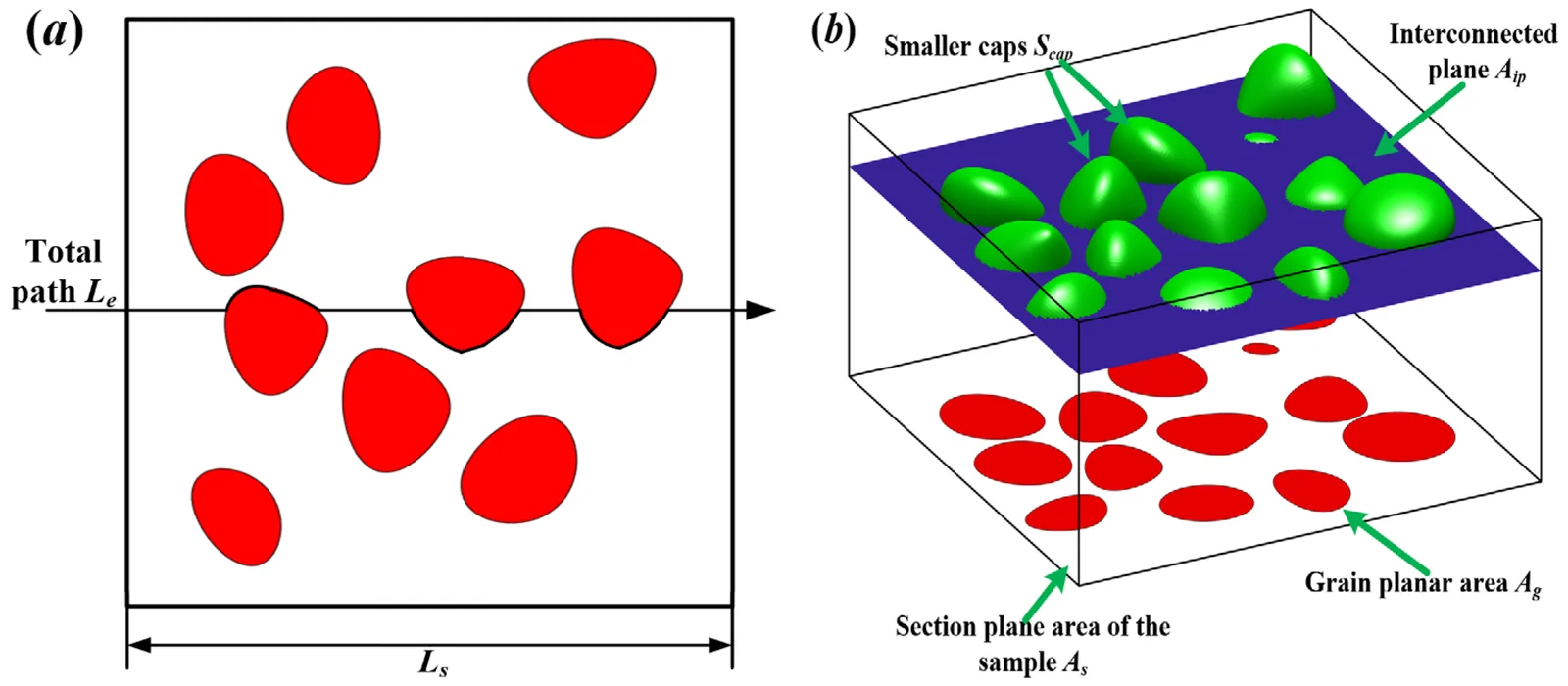
(**a**) Linear tortuosity *τ_l_* and (**b**) planar tortuosity *τ_p_*.

In the particle packing system, the linear tortuosity *τ_l_* and the planar tortuosity *τ_p_* are shown in Figure 1. The linear tortuosity *τ_l_* is the proportion of the fluid flow length to the specimen edge length. Analogous to the linear tortuosity, the planar tortuosity *τ_p_* is the ratio of the total area over which the fluid flows *S_t_* to the cross-section area of the specimen *A_s_*, i.e., the ratio of the total area of the connecting plane *A_ip_* and the surface area of each smaller cap *S_cap_* to the specimen’s cross-section area *A_s_*, as expressed by Equation (5).

(5) $$\tau_{p}=\frac{S_{e}}{A_{s}}=\frac{S_{cap}+A_{ip}}{A_{s}}=S_{A,cap}+A_{A,ip}$$

where *S_A,cap_* is the ratio of *S_cap_* to *A_s_*; *A_A,ip_* represents the connecting plane’s area fraction.

Delesse’s law [31] states that the area fraction *A_A,g_* of a component is equal to the volume fraction *V_V,g_* of that component in three-dimensional space on a random cross section. *A_A,ip_*, which refers to the proportion of the interconnecting planes’ area *A_ip_* to the specimen cross-section area *A_s_*, can be found by Equation (6).

(6) $$A_{A,ip}=\frac{A_{ip}}{A_{s}}=\frac{A_{s}-A_{g}}{A_{s}}=1-A_{A,g}=1-V_{V,g}$$

where *A_g_* is the section area of all particles that the section plan intercepts.; *A_A,g_* is the particle area fraction, which is equal to *A_g_*/*A_s_*; *V_V,g_* is the volume fraction of particles.

In order to solve for the surface area of all smaller caps *S_cap_*, one must find the average surface area of all smaller caps <*S_cap_*> and the number of particles per unit area *N_A_*. It should be remembered that *S_A_,_cap_* is equal to the product of <*S_cap_*> and *N_A_*, as stated in Equation (7).

(7) $$S_{A,cap}=N_{A}\langle S_{cap}\rangle$$

For the MPPS, assuming that the ovoidal particles of equivalent diameter *D_agg_* are arranged randomly in a cubic box with sides *L*, the possibility that the particle crossed the section is *B*_1_*D_agg_*/*L*, where *B*_1_ is the proportion of the particle caliper diameter *B* to the equivalent diameter *D_agg_*. The equivalent diameter *D_agg_* is the diameter of a corresponding sphere having the same volume as the particle under consideration. Furthermore, the number of ovoid particles that intersect the cross section *N_idp_* is equal to *B*_1_*N_agg_D_agg_*/*L*, where *N_agg_* is the quantity of ovoid particles in the box. Therefore, *N_A,m_*, the number of ovoid particles per unit area intercepted by the cross section, can be obtained by Equation (8a). Equation (8b) can be used to get the entire number of particles *N_agg_* in the box once the particle volume fraction *V_V,g_* is known. Equation (8c) displays the relationship between particle’s volume fraction *V_V,g_* and *N_A,m_*, by substituting Equation (8b) into Equation (8a).

(8a) $$N_{A,m}=\frac{N_{idp}}{L^{2}}=\frac{B_{1}N_{agg}D_{agg}}{L^{3}}=N_{V,m}D_{agg}$$

(8b) $$N_{agg}=\frac{V_{V,g}L^{3}}{V_{agg1}D_{agg}^{3}}$$

(8c) $$N_{A,m}=\frac{B_{1}N_{agg}D_{agg}}{L^{3}}=\frac{6B_{1}V_{V,g}}{\pi D_{agg}^{2}}$$

(8d) $$\begin{array}{l} B=2\int_{0}^{2\pi }d\theta \int_{\frac{-\pi }{2}}^{\frac{\pi }{2}}d\phi \{(x_{\theta }\cdot x_{\theta })[(x_{\theta }\times x_{\phi })\cdot x_{\phi \phi }]+(x_{\phi }\cdot x_{\phi })[(x_{\theta }\times x_{\phi })\cdot x_{\theta \theta }]\\ \;\;\;\;\;\;\;-2(x_{\theta }\cdot x_{\phi })[(x_{\theta }\times x_{\theta })\cdot x_{\theta \phi }]\}\times {\left[2(x_{\theta }\cdot x_{\theta })(x_{\phi }\cdot x_{\theta })-2{\left(x_{\theta }\cdot x_{\phi }\right)}^{2}\right]}^{-1}, \end{array}$$

(8e) $$x=\left[a(T\sin\theta +1)\cos\theta \cos\varphi ,b(T\sin\theta +1)\cos\theta \sin\varphi ,c\sin\theta \right]$$

(8f) $$x_{\varphi }=\left[\begin{array}{l} -a\sin\varphi \cos\theta (T\sin\theta +1)\\ \;\;b\cos\varphi \cos\theta (T\sin\theta +1)\\ \;\;0 \end{array}\right]$$

(8g) $$x_{\theta }=\left[\begin{array}{l} aT\cos\varphi \cos^{2}\theta -a\cos\varphi \sin\theta (T\sin\theta +1)\\ bT\sin\varphi \cos^{2}\theta \;-\;b\sin\varphi \;\sin\theta \;(T\sin\theta +1)\\ c\cos\theta \end{array}\right]$$

(8h) $$x_{\theta \theta }=\left[\begin{array}{l} -a\cos\varphi \cos\theta (T\sin\theta +1)-3aT\cos\varphi \cos\theta \sin\theta \\ -b\sin\varphi \cos\theta (T\sin\theta +1)\;-\;3bT\sin\varphi \cos\theta \;\sin\theta \\ -c\sin\theta \end{array}\right]$$

(8i) $$x_{\varphi \varphi }=\left[\begin{array}{l} -a\cos\varphi \cos\theta (T\sin\theta +1)\\ -b\sin\varphi \cos\theta (T\sin\theta +1)\\ 0 \end{array}\right]$$

(8j) $$x_{\theta \varphi }=\left[\begin{array}{l} a\sin\varphi \sin\theta (T\sin\theta +1)-aT\cos^{2}\theta \sin\varphi \\ bT\cos\varphi \cos^{2}\theta -b\cos\varphi \sin\theta (T\sin\theta +1)\\ 0 \end{array}\right]$$

where *V_agg_*_1_ is the proportion of a single particle’s volume *V_agg_* to the cubic equivalent diameter of a particle *D_agg_*; *V_agg_*_1_ = π/6 for the ovoid particle; *N_V,m_* is the total amount of particles per unit volume for MPPS; **x** represents the vector from the center of ovoid to the surface point *P*(*θ, φ*); **x***_θ_* and **x***_φ_* denote the first partial derivatives of **x**; **x***_θθ_*, **x***_φφ_* and **x***_θφ_* are the second partial derivatives of **x**. *θ* ∈ [−π/2, π/2] and *φ* ∈ [−π, π] of the ovoid are respectively the polar angle and azimuth angle of the point *P*(*θ, φ*) of the ellipsoid without applying tapering deformation.

After getting *N_A,m_*, which is the total number of ovoid particles per unit area intercepted by the cross-section plane, according to Equation (7), the next step is to obtain the average value of the overlap area of the fluid with all particles <*S_cap_*>. In order to determine <*S_cap_*>, a systematical cap sample method (SCSM) [32] is employed. The proportion of the average value of the overlap area of the fluid with all particles <*S_cap_*> to the particle surface area *S_agg_* is considered as the average particle crown area parameter *P_ave_*, as expressed in Equation (9). Then, <*S_cap_*> can be calculated by Equation (10).

(9) $$P_{ave}=\frac{\langle S_{cap}\rangle }{S_{agg}}$$

(10) $$\langle S_{cap}\rangle =P_{ave}S_{agg1}D_{agg}^{2}$$

(11) $$S_{agg1}=\frac{S_{agg}}{D_{agg}^{2}}$$

where the particle surface area *S_agg_* divided by the square of its equivalent diameter *D_agg_* is known as *S_agg_*_1_, as expressed by Equation (11).

The proportion of area to all minor caps *S_A,cap_* can be calculated by substituting Equations (8c)–(11) into Equation (7), as shown by Equation (12). Then, the planar tortuosity *τ_p,m_* of MPPS is established by substituting Equations (6) and (12) into Equation (5), as indicated in Equation (13).

(12) $$S_{A,capm}=N_{A,m}\langle S_{cap}\rangle =\frac{6B_{1}V_{V,g}}{\pi D_{agg}^{2}}P_{ave}S_{agg1}D_{agg}^{2}=\frac{6B_{1}V_{V,g}P_{ave}S_{agg1}}{\pi }$$

(13) $$\tau_{p,m}=1-V_{V,g}+\frac{6B_{1}V_{V,g}P_{ave}S_{agg1}}{\pi }$$

where *V_V,g_* is the volume fraction of the ovoid particle; *B*_1_ is the proportion of the particle caliper diameter to the equivalent diameter *D_agg_*; *P_ave_* is the average particle crown area parameter; *S_agg_*_1_ is the proportion of the particle surface area *S_agg_* to its equivalent diameter *D_agg_* squared.

To obtain planar tortuosity *τ_p,m_*, as indicated by Equation (13), establishing the average particle crown area parameter *P_ave_* is essential. Therefore, in this work, an SCSM is applied to obtain the average value of the overlap area of the fluid with all particles <*S_cap_*> and the average particle crown area parameter *P_ave_*.

**Figure 2:**
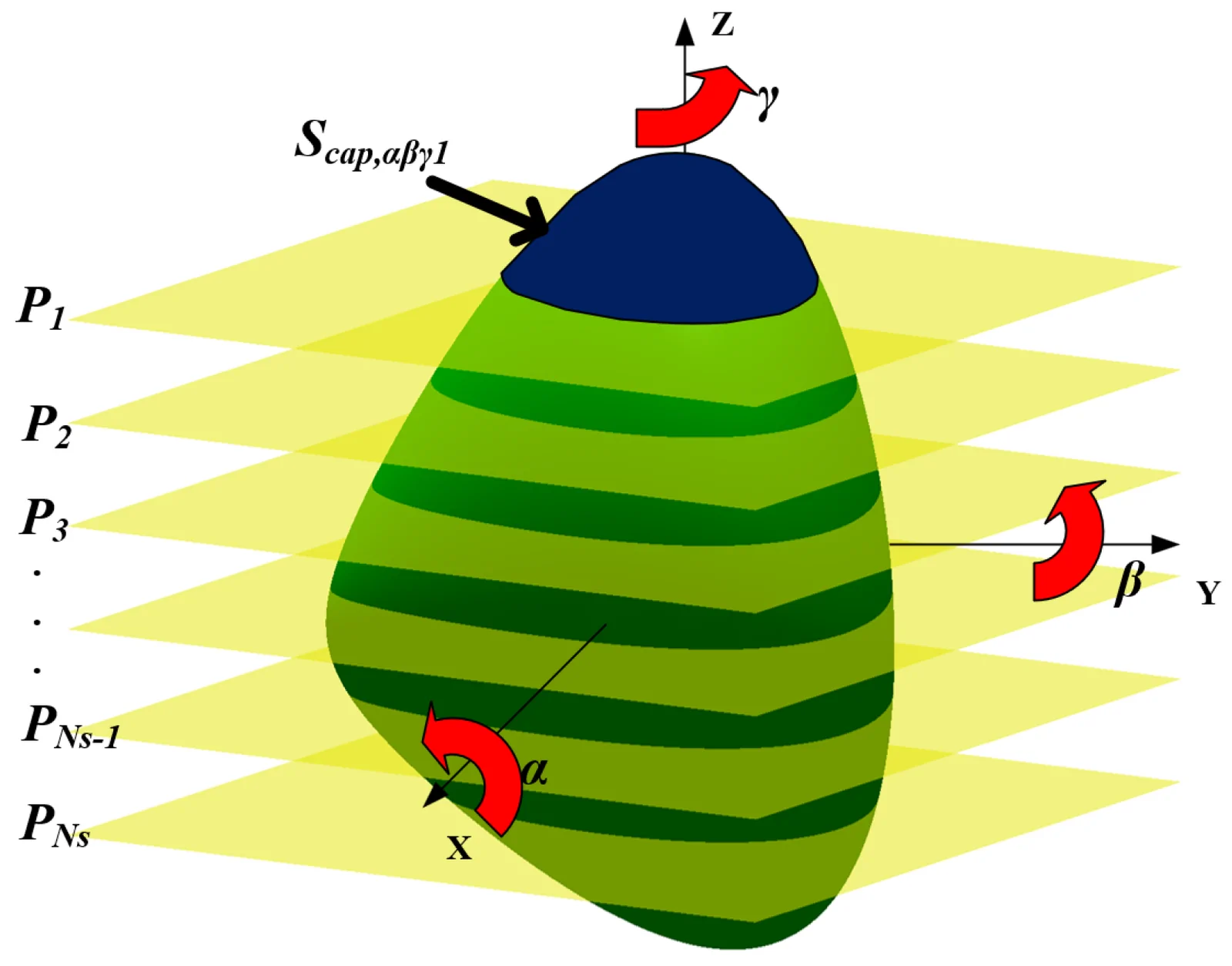
Illustration of using the SCSM to obtain the <*S_cap_*>.

To calculate the average value of the overlap surface area of the fluid with all particles <*S_cap_*>, it is necessary to take into account all potential position correlations between the section plane and particle, as displayed in Figure 2. The method accounts for the surface area of the particle in contact with the external medium *S_cap,i_* and takes a range of parallel planes *P_i_* to represent the possible positional relationships and profiles between particles, when angles of rotation *α*, *β* and *γ* are given. We average all values of *S_cap,i_*, as expressed in Equation (14). The accuracy of <*S_cap_*> is affected by the quantity of cross sections *N_s_* and the intervals of rotation ∆*α*, ∆*β* and ∆*γ*.

(14) $$\langle S_{cap}\rangle =\frac{\left[\left(\Delta \alpha +1\right)\left(\Delta \beta +1\right)\left(\Delta \gamma +1\right)\right]\sum S_{cap,\alpha \beta \gamma i}}{8\pi^{3}N_{s}}$$

where the options *α*, *β*, *γ* are the rotations corresponding to the x, y and z axes, respectively; *N_s_* denotes the quantity of cross-section planes in a particular orientation.; ∆*α*, ∆*β* and ∆*γ* are distances between two neighboring orientations (rad).

**Figure 3:**
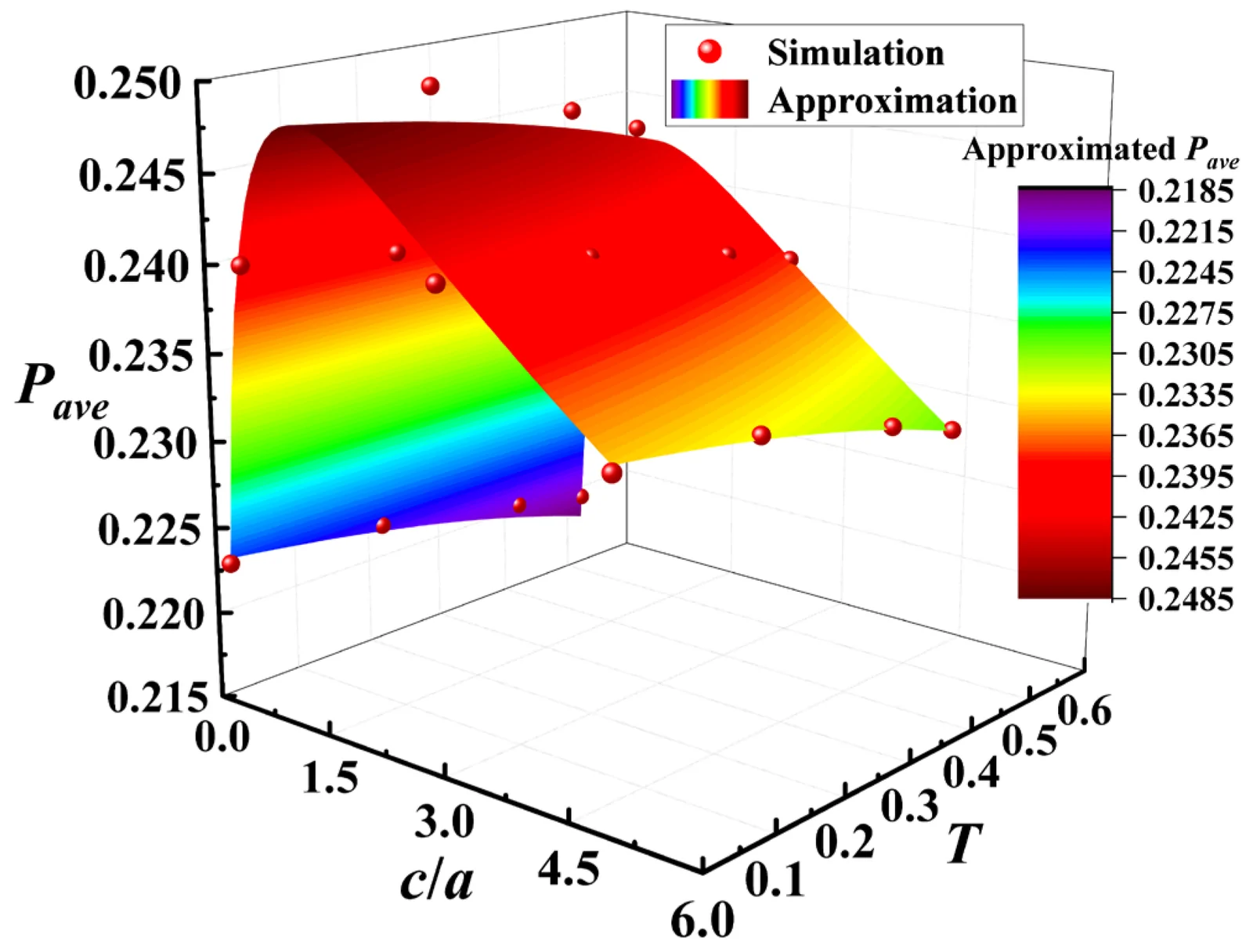
The average particle crown area parameter *P_ave_* varies for various aspect ratios *c*/*a* and tapering parameter *T.*

Through the previously mentioned SCSM, Figure 3 shows how the average particle crown area parameter *P_ave_* varies for various aspect ratios *c*/*a* and tapering parameter *T*. Findings indicate that when the tapering parameter *T* increases, *P_ave_* falls. For a given *T*, as the aspect ratio *c*/*a* grows, *P_ave_* first rises and then falls, and *P_ave_* reaches its maximum at *c*/*a* = 1.0. Nonetheless, the SCSM’s computation of *P_ave_* requires a lot more time. Therefore, in order to facilitate practical application, an approximation formula is developed, as provided by Equation (15). Here, the approximation formula exhibits excellent consistency with the numerical simulation data, achieving a high coefficient of determination R^2^ = 0.973. Moreover, the planar tortuosity *τ_p_* can be obtained by replacing the fitted average particle crown area parameter *P_ave_* from Equation (15) into Equation (13), as indicated in Equation (16).

(15) $$P_{ave}=a_{1}\ln^{a_{2}}(1+c/a)+a_{3}\exp^{2}(T)+a_{4}\ln(1+c/a)+a_{5}$$

where *a*_1_~*a*_5_ are fitting parameters, which can be found in Table 1.

(16) $$\tau_{p,m}=1-V_{V,g}+\frac{6B_{1}V_{V,g}(a_{1}\ln^{a_{2}}(1+c/a)+a_{3}\exp(2T)+a_{4}\ln(1+c/a)+a_{5})S_{agg1}}{\pi }$$

### 2.2. Poly-Sized Ovoid Particle Packing System (PPPS)

Similar to MPPS, according to Equation (8), *N_A,p_*(*D_agg_*) is the number of ovoid particles with equivalent diameter *D_agg_* per unit area intercepted by the cross section intercepted for PPPS, and *N_V,p_*(*D_agg_*) is the number of ovoid particles with equivalent diameter *D_agg_* per unit volume of container intercepted by the cross section intercepted for PPPS, as shown in Equation (17). *N_V,p_*(*D_agg_*) and the probability density function of the volume-based size distribution *f_V,size_*(*D_agg_*) are related, as demonstrated by Formula (18). In addition to *f_V,size_*(*D_agg_*), the probability density function of the number-based size distribution *f_N,size_*(*D_agg_*) is also used in theoretical and numerical calculations. The numerical correlation between *f_V,size_*(*D_agg_*) and *f_N,size_*(*D_agg_*) is expressed by Equation (19). The relationship between *N_A,p_*(*D_agg_*) and the probability density function of number-based *f_N,size_*(*D_agg_*) is established by substituting Equations (18) and (19) into Equation (17), as expressed in Equation (20).

(17) $$N_{A,p}(D_{agg})=D_{agg}N_{V,p}(D_{agg})$$

(18) $$N_{V,p}(D_{agg})=\frac{B_{1}V_{V,g}f_{V,size}(D_{agg})dD_{agg}}{V_{agg1}D_{agg}^{3}}$$

(19) $$f_{V,size}(D_{agg})dD_{agg}=\frac{V_{agg1}D_{agg}^{3}f_{N,size}(D_{agg})dD_{agg}}{\int_{D_{agg,\min}}^{D_{agg,\max}}f_{N,size}(D_{agg})V_{agg1}D_{agg}^{3}dD_{agg}}$$

(20) $$N_{A,p}(D_{agg})=\frac{B_{1}V_{V,g}D_{agg}f_{N,size}(D_{agg})dD_{agg}}{\int_{D_{agg,\min}}^{D_{agg,\max}}f_{N,size}(D_{agg})V_{agg1}D_{agg}^{3}dD_{agg}}$$

where *N_A,p_*(*D_agg_*) is the number of ovoid particles with equivalent diameter *D_agg_* per unit area intercepted by the cross section intercepted for PPPS; *f_V,size_*(*D_agg_*) is the probability density function of the volume-based size distribution; *f_N,size_*(*D_agg_*) is the probability density function of the number-based particle size distribution; *B*_1_ is the proportion of the particle caliper diameter to the equivalent diameter *D_agg_*.

**Figure 4:**
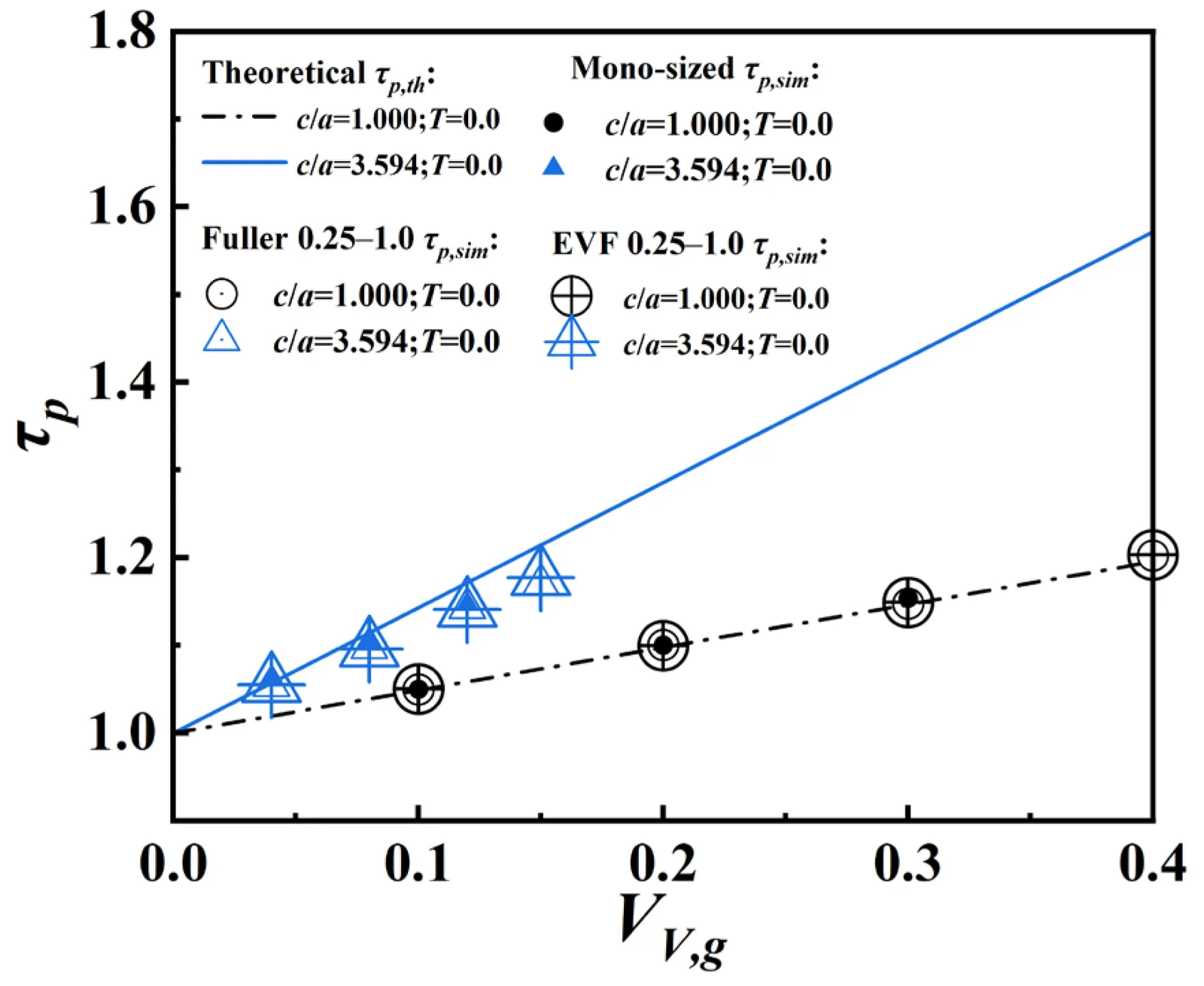
Comparison of *τ_p_* from the theoretical model with those from numerical simulations [32].

The area fraction of all smaller caps for the PPPS *S_A,capp_* is the integration of the multiplication of *N_A,p_*(*D_agg_*) and <*S_cap_*(*D_agg_*)>, as expressed by Equation (21), and can be obtained by substituting <*S_cap_*> in Equation (10) and *N_A,p_*(*D_agg_*) in Equation (20) into Equation (21), as indicated in Equation (22). Substituting *A_A,ip_* in Equation (6) and *S_A,capp_* in Equation (22) into Equation (5), the planar tortuosity *τ_p,p_* of PPPS can be calculated, as indicated in Equation (23a). It can be revealed that the planar tortuosity *τ_p_* is irrelevant to the particle size distribution. To verify the reliability of the tortuosity theory, Figure 4 presents a comparison between the numerical data from Li et al. [32] and the theoretical tortuosity *τ_p_* derived from Equation (23a). For analyzing the size distribution of the particles, the Fuller function as stated in Equation (23b) and the Equivalent Volume Function as displayed in Equation (23c) are used. Figure 4 presents consistent agreement between the theoretical tortuosity *τ_p_* and numerical simulation data, the coefficient of determination for particles *c*/*a* = 1.000, *T* = 0.0 is R^2^ = 0.9954 and for *c*/*a* = 3.594, *T* = 0.0 is R^2^ = 0.6811. The proposed tortuosity model exhibits nearly perfect consistency with the data for near-spherical ovoids, while its predictive capacity deteriorates significantly for elongated particles. The weak fitting degree for elongated ovoids arises from narrow constricted pore throats and abundant stagnant flow regions generated by slender particle random packing, which break the stereological geometric assumptions underpinning our planar tortuosity derivation. Moreover, Figure 4 suggests that the tortuosity *τ_p,m_* value of MPPS is similar to those *τ_p,p_* of PPPS, which demonstrates that the planar tortuosity is irrelevant to the particle size distribution.

(21) $$S_{A,capp}=\int_{D_{agg,\min}}^{D_{agg,\max}}N_{A,p}(D_{agg})\langle S_{cap}(D_{agg})\rangle dD_{agg}$$

(22) $$S_{A,capp}=\int_{D_{agg,\min}}^{D_{agg,\max}}\frac{B_{1}V_{V,g}P_{ave}S_{agg1}D_{agg}^{3}f_{N,size}(D_{agg})}{\int_{D_{agg,\min}}^{D_{agg,\max}}f_{N,size}(D_{agg})V_{agg1}D_{agg}^{3}dD_{agg}}dD_{agg}=\frac{6B_{1}V_{V,g}P_{ave}S_{agg1}}{\pi }$$

(23a) $$\tau_{p,p}=1-V_{V,g}+\frac{6B_{1}V_{V,g}P_{ave}S_{agg1}}{\pi }$$

(23b) $$f_{N,sizeFuller}(D_{agg})=\frac{5D_{agg}^{-3.5}}{2(D_{agg,\min}^{-2.5}-D_{agg,\max}^{-2.5})}$$

(23c) $$f_{N,sizeEVF}(D_{agg})=\frac{3D_{agg}^{-4}}{\left(D_{agg,\min}^{-3}-D_{agg,\max}^{-3}\right)}$$

where *S_A,capp_* is the area fraction of all small caps for PPPS; *P_ave_* is the average particle crown area parameter; *S_agg_*_1_ is the particle surface area *S_agg_* divided by the square of its equivalent diameter *D_agg_*; *τ_p,p_* is the planar tortuosity for PPPS.

**Figure 5:**
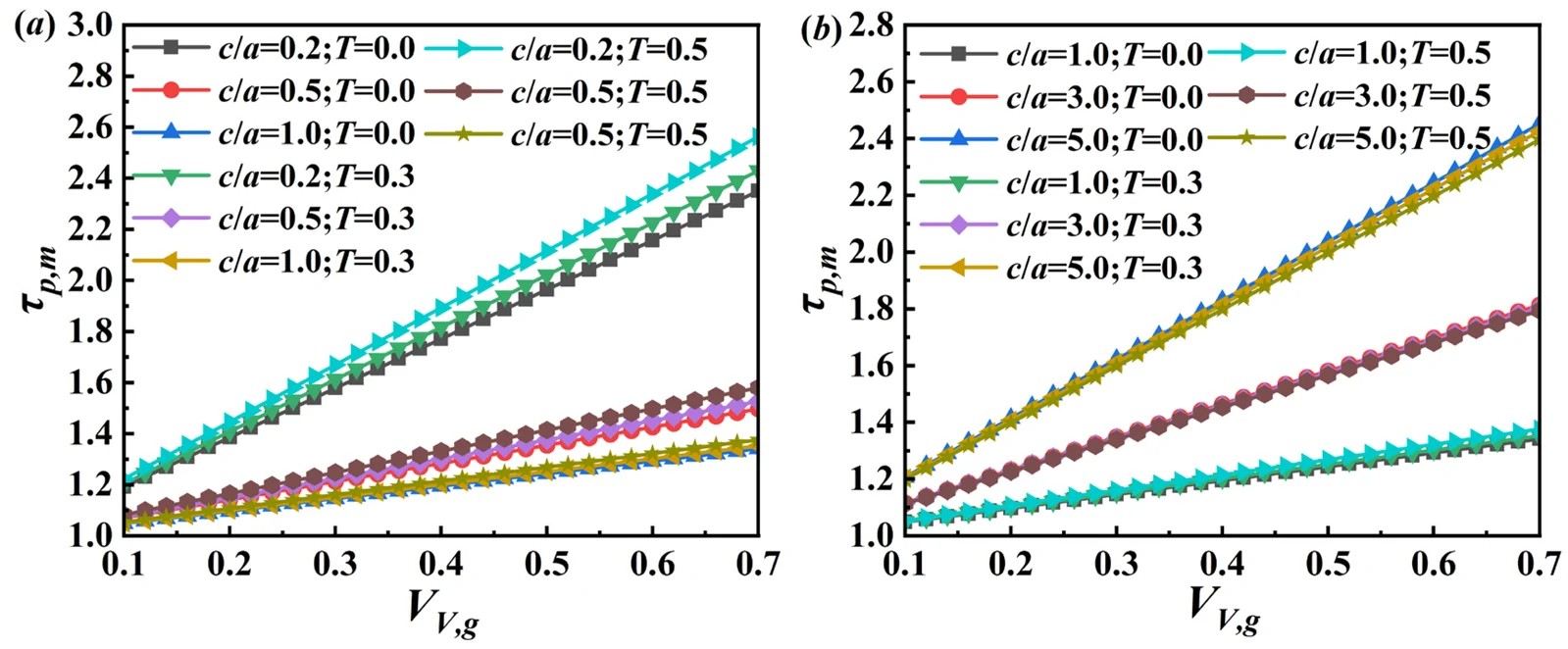
Effect of grain shape on geometric tortuosity *τ_p,m_*: (**a**) oblate ovoid and (**b**) prolate ovoid.

Considering the previously mentioned theoretical model of planar tortuosity, Figure 5 shows how the tortuosity–volume fraction curves vary with the aspect ratio *c*/*a* and tapering parameter *T*. The results demonstrate that when the volume fraction rises, the tortuosity increases. When tapering parameter *T* and the volume fraction are given, with an aspect ratio of *c*/*a* greater than 1.0, the tortuosity *τ_p,m_* of the particle packing system grows; when the particle aspect ratio *c*/*a* is less than 1.0, the tortuosity *τ_p,m_* of the particle packing system falls as *c*/*a* increases, and the tortuosity *τ_p,m_* of *c*/*a* = 1.0 and *T* = 0.0 has minimal value.

## 3. The Effect of Ovoid Particle Composition on the K-C Formula

To investigate how the permeability *k* of the particle packing system is affected by the particle’s volume fraction and shape, the lattice Boltzmann method (LBM) is employed. The LBM simulates the fluid flow in granular media and can be applied for the solution of the permeability *k* by discretizing complicated particles into simple lattices. Moreover, it should be noted that *τ_p_* is planar geometrical tortuosity, which differs distinctly from hydraulic tortuosity. Planar tortuosity is quantified based on the stereological area ratio of pore cross sections and characterizes the geometric feature of pore networks from a sectional perspective. Hydraulic tortuosity, by contrast, describes the winding length of actual fluid streamlines and is calculated from flow velocity fields. These two parameters correspond to different physical characterization dimensions of porous media pore structures.

### 3.1. The Lattice Boltzmann Method

**Figure 6:**
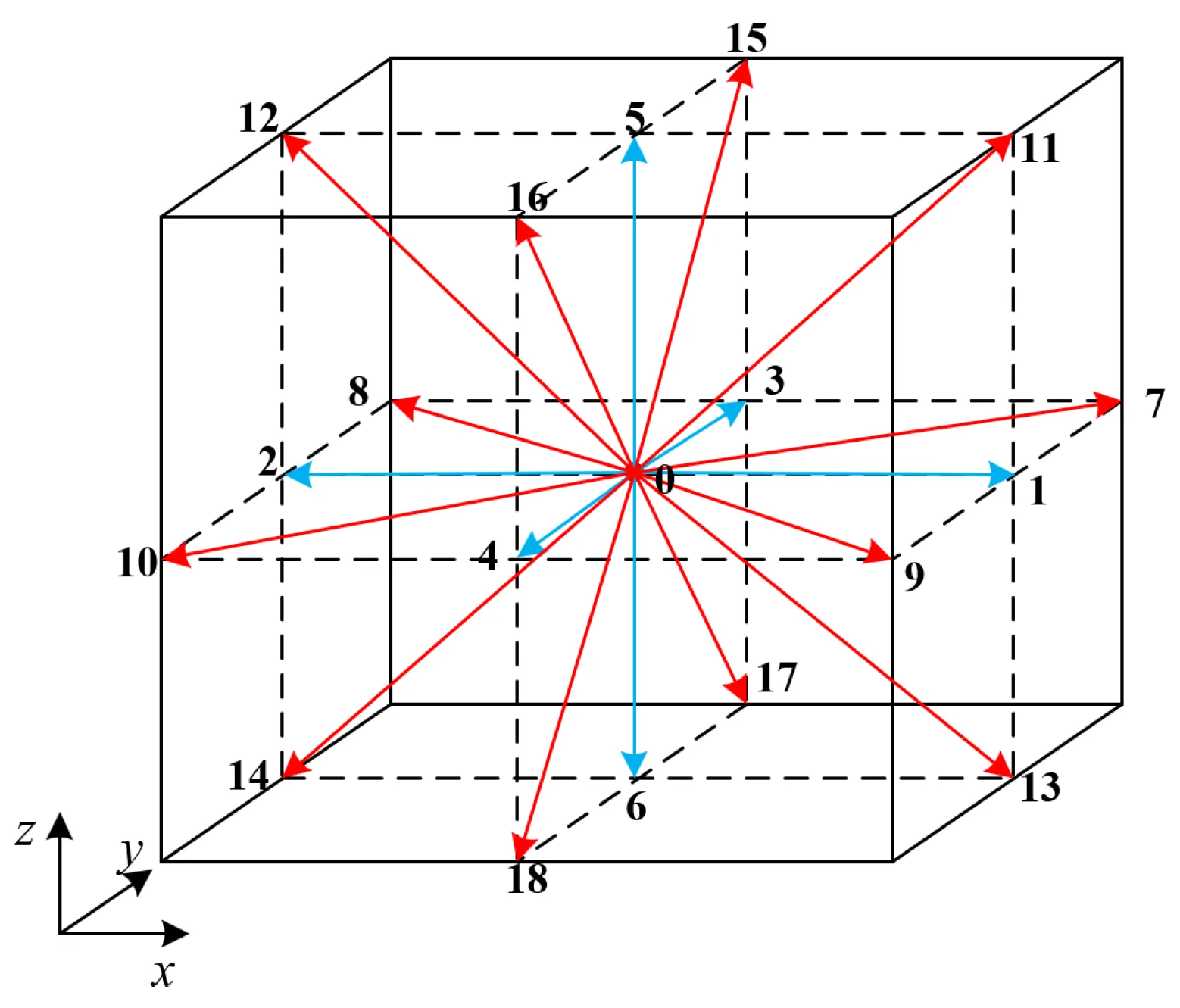
Illustration of the D3Q19 model for LBM.

As shown in Figure 6, the particles in this work are discretized using a three-dimensional D3Q19 lattice with 19 velocities. Under the LBM condition, the collision propagation formula is formulated as Equation (24a).

(24a) $$f_{\alpha }\left(x+e_{\alpha }\Delta t,t+\Delta t\right)=f_{\alpha }\left(x,t\right)-\frac{1}{\tau_{rel}}\left[f_{\alpha }\left(x,t\right)-f_{\alpha }^{eq}\left(x,t\right)\right]$$

where *f_α_*(*x*,*t*) is the particle probability distribution function with speed ***e***_α_ at point **x** and time *t*; ***e***_α_ represents the microscopic speed vector that corresponds to allowable directions on the lattice, as given by Equation (24b). The number of allowable speed directions is denoted by the subscript *α*. According to Equation (24c), *τ_rel_* represents the dimensionless relaxation time; *f_α_^eq^*(*x*,*t*) is the equilibrium particle probability distribution function, as displayed in Equation (24d).

(24b) $$e_{\alpha }=\left[\begin{array}{ccccccccccccccccccc} 0 & 1 & -1 & 0 & 0 & 0 & 0 & 1 & -1 & 1 & -1 & 1 & -1 & 1 & -1 & 0 & 0 & 0 & 1\\ 0 & 0 & 0 & 1 & -1 & 0 & 0 & 1 & -1 & -1 & 1 & 0 & 0 & 0 & 0 & 1 & -1 & 1 & -1\\ 0 & 0 & 0 & 0 & 0 & 1 & -1 & 0 & 0 & 0 & 0 & 1 & -1 & -1 & 1 & 1 & -1 & -1 & 1 \end{array}\right]$$

(24c) $$\tau_{rel}=\frac{k_{v}}{\delta tc_{s}^{2}}+\frac{1}{2}$$

(24d) $$f_{\alpha }^{eq}(x,t)=\rho w_{\alpha }\left[1+\frac{e_{\alpha }\cdot u}{c_{s}^{2}}+\frac{{\left(e_{\alpha }\cdot u\right)}^{2}}{2c_{s}^{4}}-\frac{u^{2}}{2c_{s}^{2}}\right]$$

where the density of the fluid, represented by *ρ* (mu/lu^3^), can be determined by Equation (25a); *k_v_* represents the flow’s kinetic viscosity (lu^2^/ts), and with a value of 1/6, it causes the relaxed time *τ_rel_* = 1; *c_s_* is the lattice rate of sound (lu/ts), which has the value (1/3)^1/2^ (∆x/*δ_t_*) [33]; *δ_t_* is the time step (ts), and *δ_t_* = 1 ts; *w* means the weight factor (*w*_0_ = 1/3 for *α* = 0; *w*_0_ = 1/18 for *α* = 1~6; *w*_0_ = 1/36 for *α* = 7~18); **u** is the speed (lu/ts), which is calculated using Equation (25b).

(25a) $$\rho =\rho \left(x,t\right)=\sum_{\alpha }f_{\alpha }\left(x,t\right)$$

(25b) $$u=u\left(x,t\right)=\frac{1}{\rho }\sum_{\alpha }f_{\alpha }e_{\alpha }$$

To investigate the particle permeability in this work, three different border conditions (i.e., the input/output pressure border condition, the half-way bounce-back border condition and the periodic border condition) are adopted. Firstly, the input and output pressure border conditions are subject to the non-equilibrium bounce-back rule. [34]. If the input and output borders are perpendicular to the x-direction, and both the output density (*ρ_out_*) and the input density (*ρ_in_*) are fixed, following the transmission process, the components of the distribution function at the input border (i.e., *f*_1_, *f*_7_, *f*_9_, *f*_11_, *f*_13_) or output border (i.e., *f*_2_, *f*_8_, *f*_10_, *f*_12_, *f*_14_) are uncertain. From the remaining previously identified components, the unidentified components can be achieved, as shown in Equations (26) and (27). Afterwards, in order to identify the unidentified components of the particle distribution functions of the nodes of opposite borders from the known components, the periodic boundary condition is immediately applied to the remaining box edges. Under the circumstances, the function components that leave one border will travel straight to the contrary border. In addition, when dealing with the flow–solid boundary, the half-way bounce-back border rule is used in this work. It is presumed that a solid wall is situated halfway between the fluid and solid nodes in this instance. The distribution functions scatter from the adjacent rigid nodes where the fluid is flowing back to the flow node along the direction of movement.

In the input border (i.e., left border):

(26a) $$u_{x,in}=1-\frac{1}{\rho_{in}}\left[\begin{array}{l} f_{3}+f_{4}+f_{5}+f_{6}+f_{15}+f_{16}+f_{17}+f_{18}+f_{0}\\ +2\left(f_{2}+f_{8}+f_{10}+f_{12}+f_{14}\right) \end{array}\right]$$

(26b) $$f_{1}=f_{2}+\frac{1}{3}\rho_{in}u_{x,in}$$

(26c) $$f_{7}=f_{10}+\frac{1}{6}\rho_{in}u_{x,in}-\frac{1}{2}\left[f_{3}+f_{15}+f_{17}-\left(f_{4}+f_{16}+f_{18}\right)\right]$$

(26d) $$f_{9}=f_{8}+\frac{1}{6}\rho_{in}u_{x,in}-\frac{1}{2}\left[f_{3}+f_{15}+f_{17}-\left(f_{4}+f_{16}+f_{18}\right)\right]$$

(26e) $$f_{11}=f_{14}+\frac{1}{6}\rho_{in}u_{x,in}-\frac{1}{2}\left[f_{5}+f_{15}+f_{16}-\left(f_{6}+f_{17}+f_{18}\right)\right]$$

(26f) $$f_{13}=f_{12}+\frac{1}{6}\rho_{in}u_{x,in}-\frac{1}{2}\left[f_{5}+f_{15}+f_{16}-\left(f_{6}+f_{17}+f_{18}\right)\right]$$

(26g) $$p_{in}=c_{s}^{2}\rho_{in}=\frac{1}{3}\rho_{in}$$

In the output border (i.e., right border):

(27a) $$u_{x,out}=-1+\frac{1}{\rho_{out}}\left[\begin{array}{l} f_{3}+f_{4}+f_{5}+f_{6}+f_{15}+f_{16}+f_{17}+f_{18}+f_{0}\\ +2\left(f_{1}+f_{7}+f_{9}+f_{11}+f_{13}\right) \end{array}\right]$$

(27b) $$f_{2}=f_{1}-\frac{1}{3}\rho_{out}u_{x,out}$$

(27c) $$f_{8}=f_{9}-\frac{1}{6}\rho_{out}u_{x,out}-\frac{1}{2}\left[f_{3}+f_{15}+f_{17}-\left(f_{4}+f_{16}+f_{18}\right)\right]$$

(27d) $$f_{10}=f_{7}-\frac{1}{6}\rho_{out}u_{x,out}-\frac{1}{2}\left[f_{3}+f_{15}+f_{17}-\left(f_{4}+f_{16}+f_{18}\right)\right]$$

(27e) $$f_{12}=f_{13}-\frac{1}{6}\rho_{out}u_{x,out}-\frac{1}{2}\left[f_{5}+f_{15}+f_{16}-\left(f_{6}+f_{17}+f_{18}\right)\right]$$

(27f) $$f_{14}=f_{11}-\frac{1}{6}\rho_{out}u_{x,out}-\frac{1}{2}\left[f_{5}+f_{15}+f_{16}-\left(f_{6}+f_{17}+f_{18}\right)\right]$$

(27g) $$\rho_{out}=c_{s}^{2}\rho_{out}=\frac{1}{3}\rho_{out}$$

To simulate fluid flow, there is a pressure difference (i.e., x-direction) at the sample input and outflow in the LBM simulation. The nodes on the opposite side of the border are regarded as neighbors, and the other sides are regarded as periodic borders. For the flow–solid interface, the half-way bounce-back borders were applied. Equation (28) provides the equilibrium flow standards for speed *u_x_* across an entire region. If the aforementioned formula is satisfied, the permeability *k* will be determined easily. The characteristic permeability of the LBM *k_LB_* may be computed by employing Equation (29) provided that the steady-state flow requirement is met, and the value of the pressure variation is oriented in the *x*-axis. Next, a correspondence between actual mechanical units and LBM units establish the link between the permeability *k* of the real physical system and the permeability *k_LB_* of LBM [33], as indicated in Equation (30).

(28) $$\sqrt{\frac{{\sum \left[u_{x}\left(t+\delta t\right)-u_{x}\left(t\right)\right]}^{2}}{\sum u_{x}{\left(t+\delta t\right)}^{2}}}\leq 1.0\times 10^{-5}$$

(29) $$k_{LB}=\frac{k_{v}}{\Delta P}\langle u_{x}\rangle =\frac{k_{v}L_{1}}{P_{in}-P_{out}}\langle u_{x}\rangle$$

(30) $$k=k_{LB}L_{0}^{2}$$

where <*u_x_*> represents the average speed in the *x*-axis (lu/ts); *L*_0_ represents the size of the mapping between real material units and LBM units (m^2^/lu^2^); *P_in_* and *P_out_* represent the pressures at the border of the intake and outflow (mu/(lu*ts^2^)), respectively; *L*_1_ denotes the border’s length of the cubed sample.

All numerical tests adopt a cubic computational domain with uniform side length *L* = 100 μm, D3Q19 LBM mesh resolution 100 × 100 × 100, and single lattice step Δx = 1 μm. The ovoid geometric variable ranges are as follows: aspect ratio *c*/*a* ∈ [0.2,5.0], tapering parameter *T* ∈ [0,0.5]. For each fixed parameter combination, 10 independent Monte Carlo spatial and rotational random realizations are simulated to calculate the mean values and standard deviation as statistical uncertainty. All packing assemblies strictly prohibit inter-particle overlap, and the sampling workflow contains following fixed steps. Randomly sample mass center coordinates inside the cubic domain for each ovoid particle; conduct uniform Euler angle rotation sampling on x/y/z axes to determine the particle spatial orientation; implement inter-particle surface distance detection; discard and re-sample overlapping coordinate/orientation groups; iterate sampling until the target volume fraction converges with a relative error less than 0.1%.

### 3.2. Modified K-C Formula

According to Equations (3) and (4), it can be determined that the shape parameter *C_f_* and tortuosity *τ* have an impact on the K-C constant *C_k_*. Regarding the tortuosity, the influence of particle shape and volume fraction on the planar tortuosity *τ_p_* are quantitatively characterized in Section 2’s tortuosity theoretical model; however, it is unknown that how these factors affect *C_f_*, which is not beneficial for the performance of the K-C formula. In order to derive *C_k_*, the permeability *k* from LBM is first inserted into Equation (31). Substituting the K-C constant *C_k_* and the planar tortuosity *τ_p_* by the theoretical model into Equation (32) obtains shape parameter *C_f_*.

(31) $$C_{k}=\frac{{\left(1-V_{V,g}\right)}^{3}}{kS_{V,g}^{2}V_{V,g}^{2}}$$

(32) $$C_{f}=\frac{C_{k}}{\tau_{p}^{2}}$$

**Figure 7:**
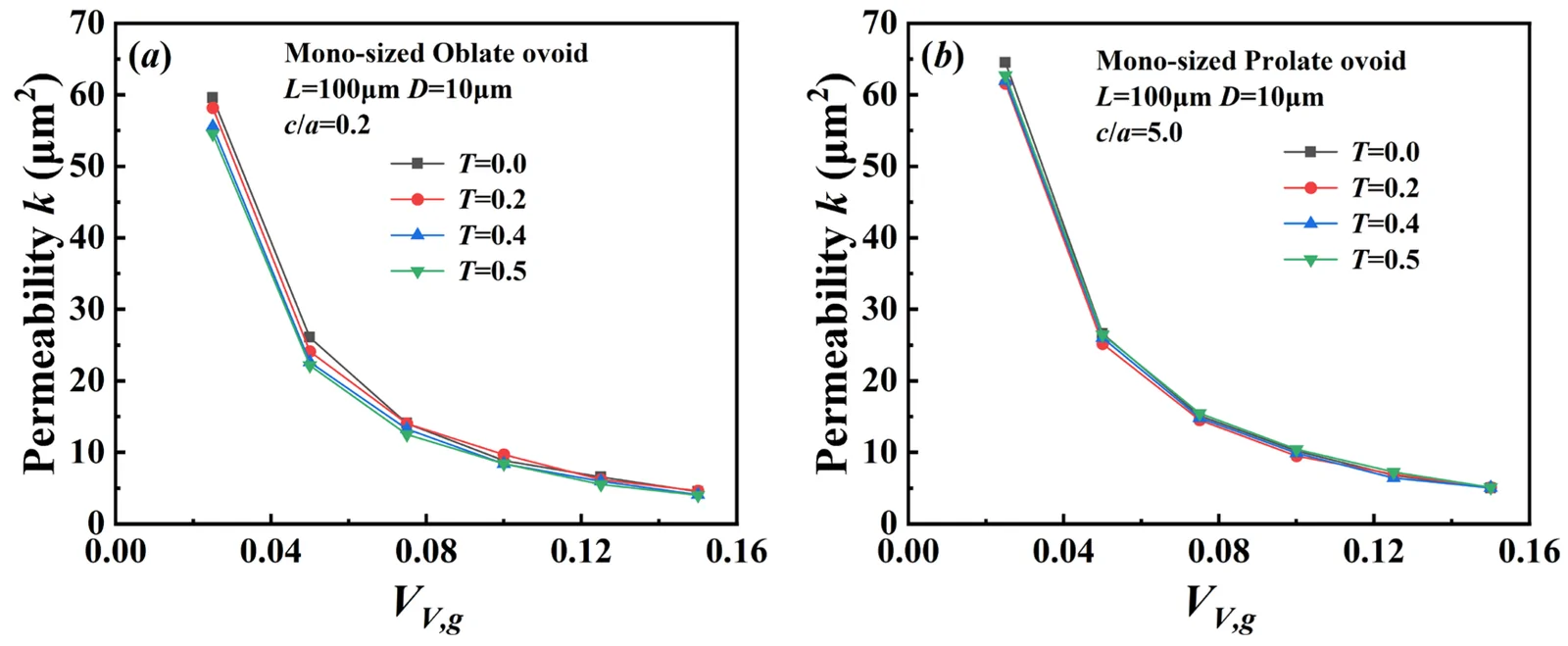
Effect of *T* on permeability *k*: (**a**) particle with *c*/*a* = 0.2; (**b**) particle with *c*/*a* = 5.0.

**Figure 8:**
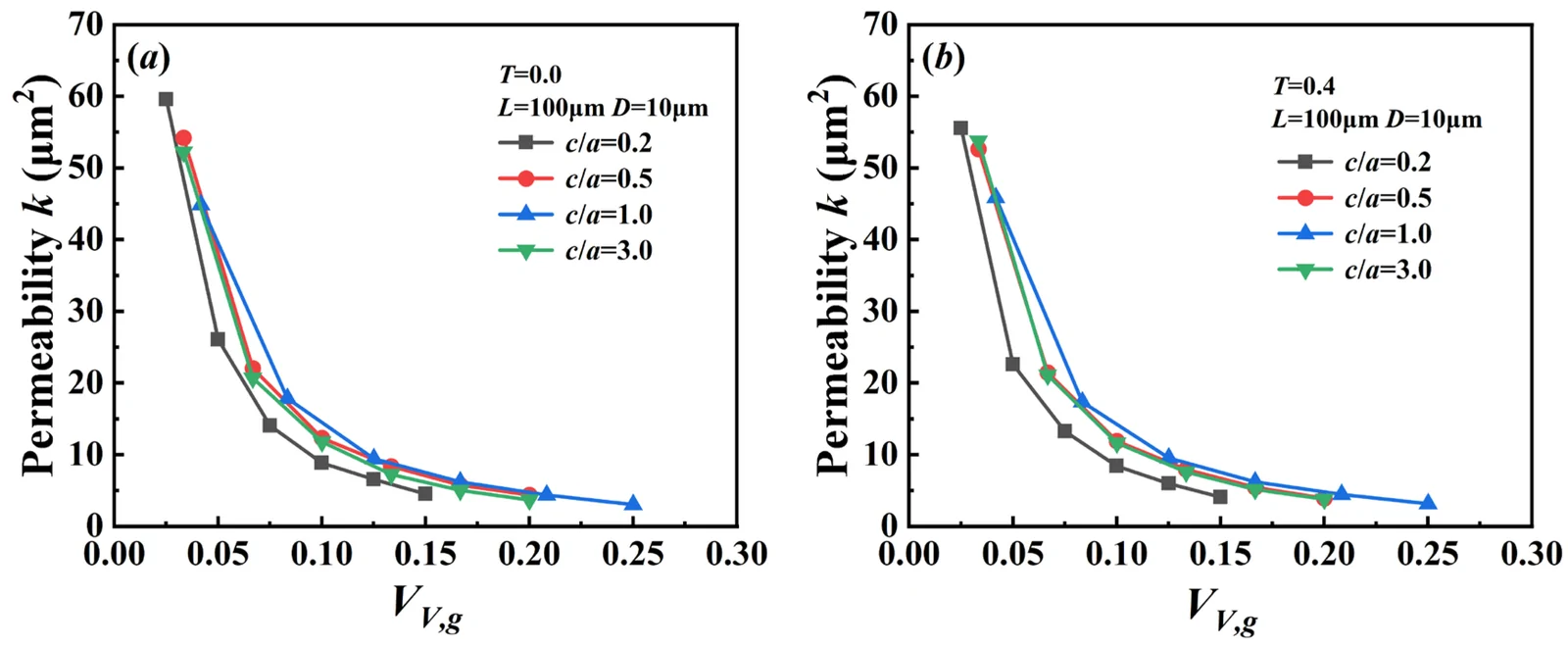
Effect of *c*/*a* on permeability *k*: (**a**) particle with *T* = 0.0; (**b**) particle with *T* = 0.4.

We explore how the volume fraction and shape of the ovoidal particles affect shape parameter *C_f_* and the permeability *k* of the porous composites. We also investigate the effect of the ovoidal particle shape and volume fraction on the permeability *k* and the shape parameter *C_f_* of the porous composites. The permeability *k* of the particle packing system was obtained according to the LBM model as shown in Figure 7 and Figure 8. The permeability *k* is observed to decrease as the volume fraction increases. Given the tapering parameter *T* and volume fraction *V_V,g_*, the permeability *k* first rises and subsequently falls as the aspect ratio *c*/*a* increases, with the aspect ratio *c*/*a* = 1.0, the maximum permeability is achieved.

**Figure 9:**
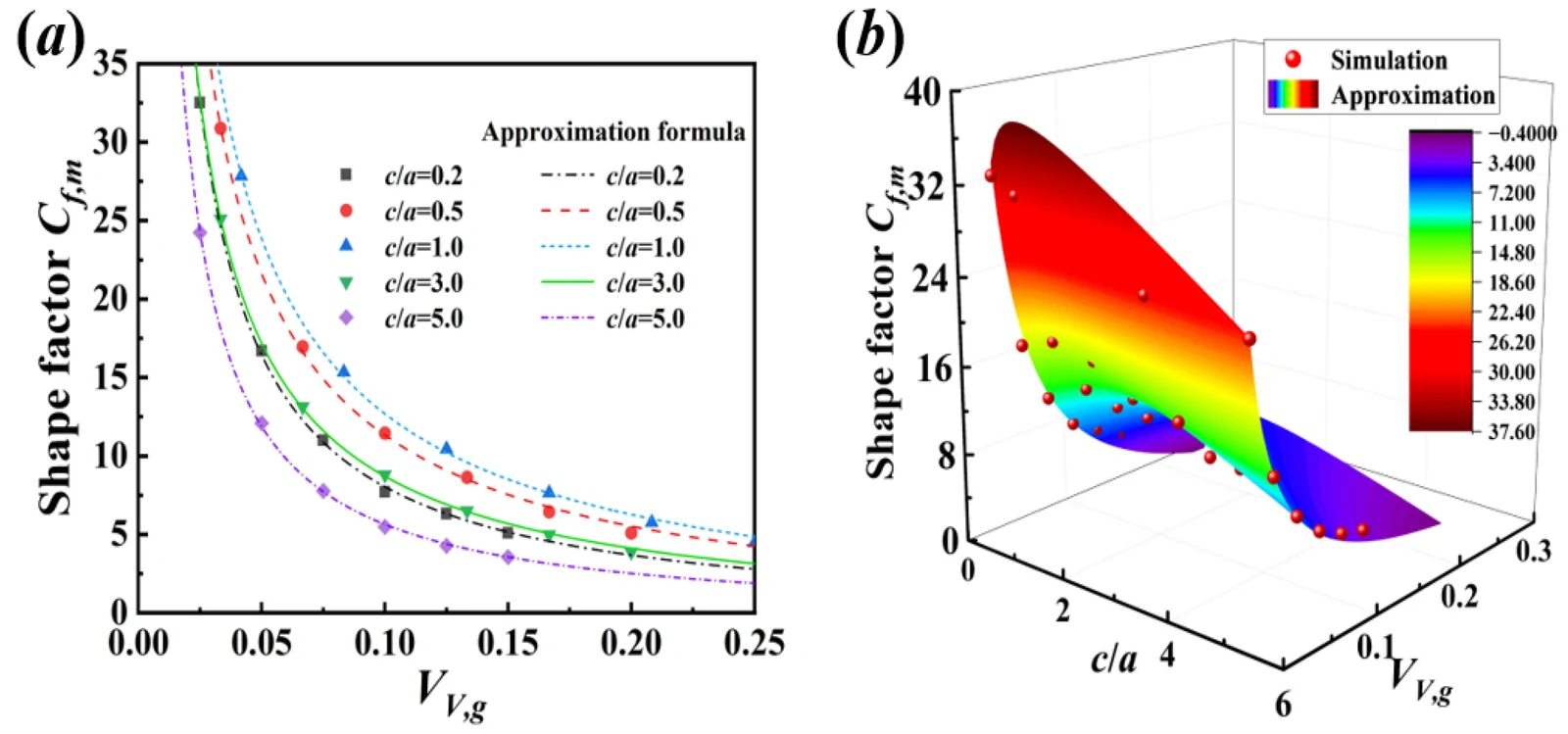
(**a**) Effect of grain shape on shape factor *C_f,m_*. (**b**) Approximation of shape factor *C_f,m_*.

**Figure 10:**
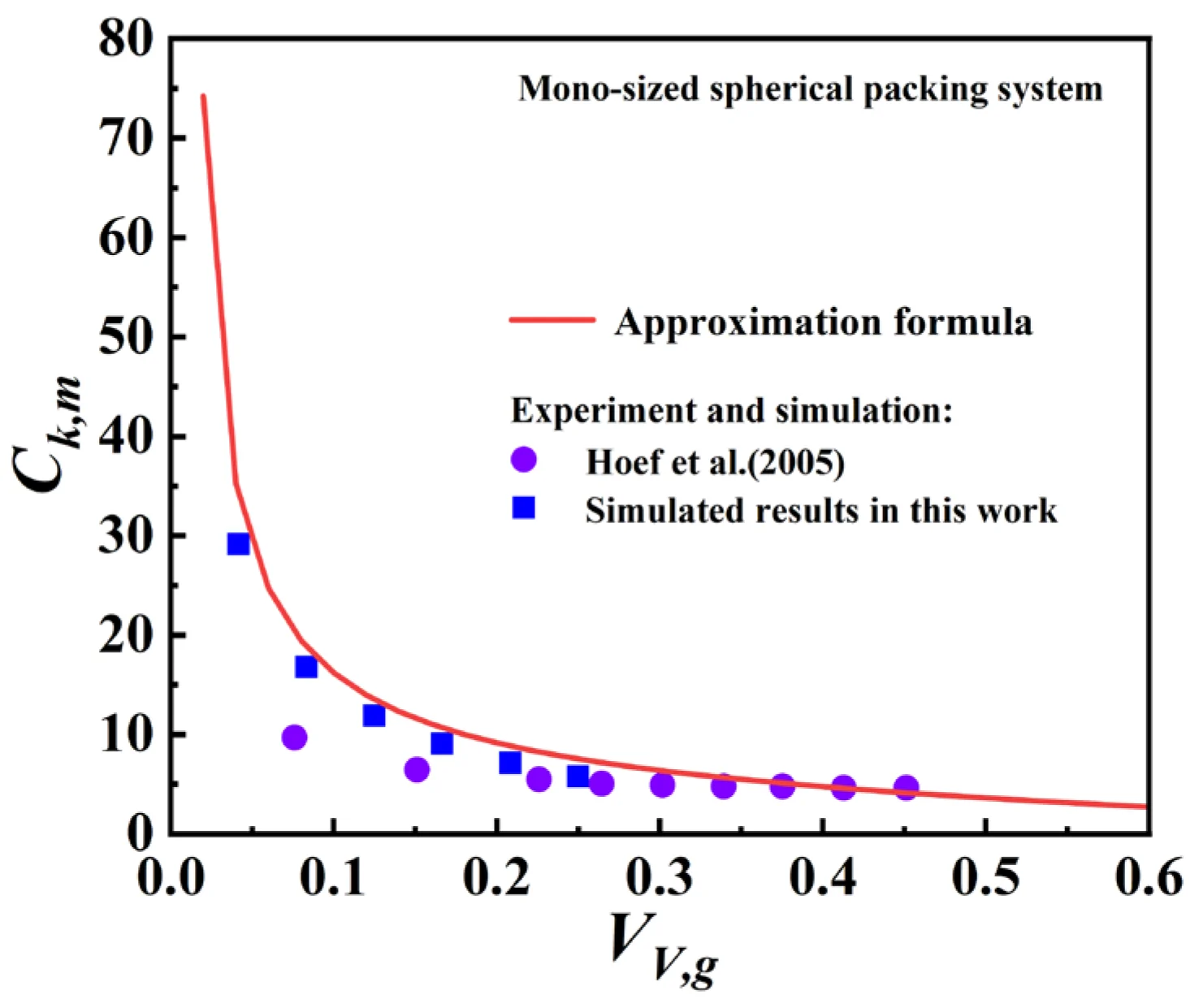
Comparison of the approximation for *C_k,m_* with other studies [36].

By substituting the permeability *k* from Figure 8 into Equations (31) and (32), the shape parameter *C_f_* was obtained. Figure 9 indicates the variation in the shape parameter *C_f,m_* with the volume fraction *V_V,g_* in the MPPS, and the results show that the shape parameter *C_f,m_* is not constant and instead declines as the particle volume fraction *V_V,g_* increases. An approximate formula is proposed in this work, as shown in Equation (33), which can characterize quantitatively the influence of the aspect ratio *c*/*a* and volume fraction *V_V,g_* of the ovoid particles on the shape parameter C_f_. Figure 9 compares the shape parameters *C_f,m_* obtained from the fitted formulation and the simulation; the fitted expression of *C_f_* achieves a determination coefficient R^2^ = 0.9872, which shows good agreement between both values. Then, the K-C formula constants *C_k,m_* for mono-sized particles can be found by substituting the tortuosity *τ_p_* from Equation (13) and the shape parameter *C_f_* obtained in Equation (33) into Equation (4). To confirm the credibility of the obtained approximation of *C_k,m_*, Figure 10 compares the approximated *C_k,m_* derived in Equations (4), (14) and (33) with the simulated *C_k,m_* in this work and preceding investigations [35,36]. The results demonstrate that the approximation of *C_k,m_* agrees well with the simulation results, which confirms the reliability of the formulation proposed in this paper for the calculation of the K-C formula constant *C_k,m_*.

(33) $$C_{f,m}=-3.41{\left(c/a\right)}^{-0.65}+4.55V_{V,g}^{-0.63}-1.06(c/a)V_{V,g}^{-0.33}$$

where *C_f,m_* is the shape parameter of the MPPS.

**Figure 11:**
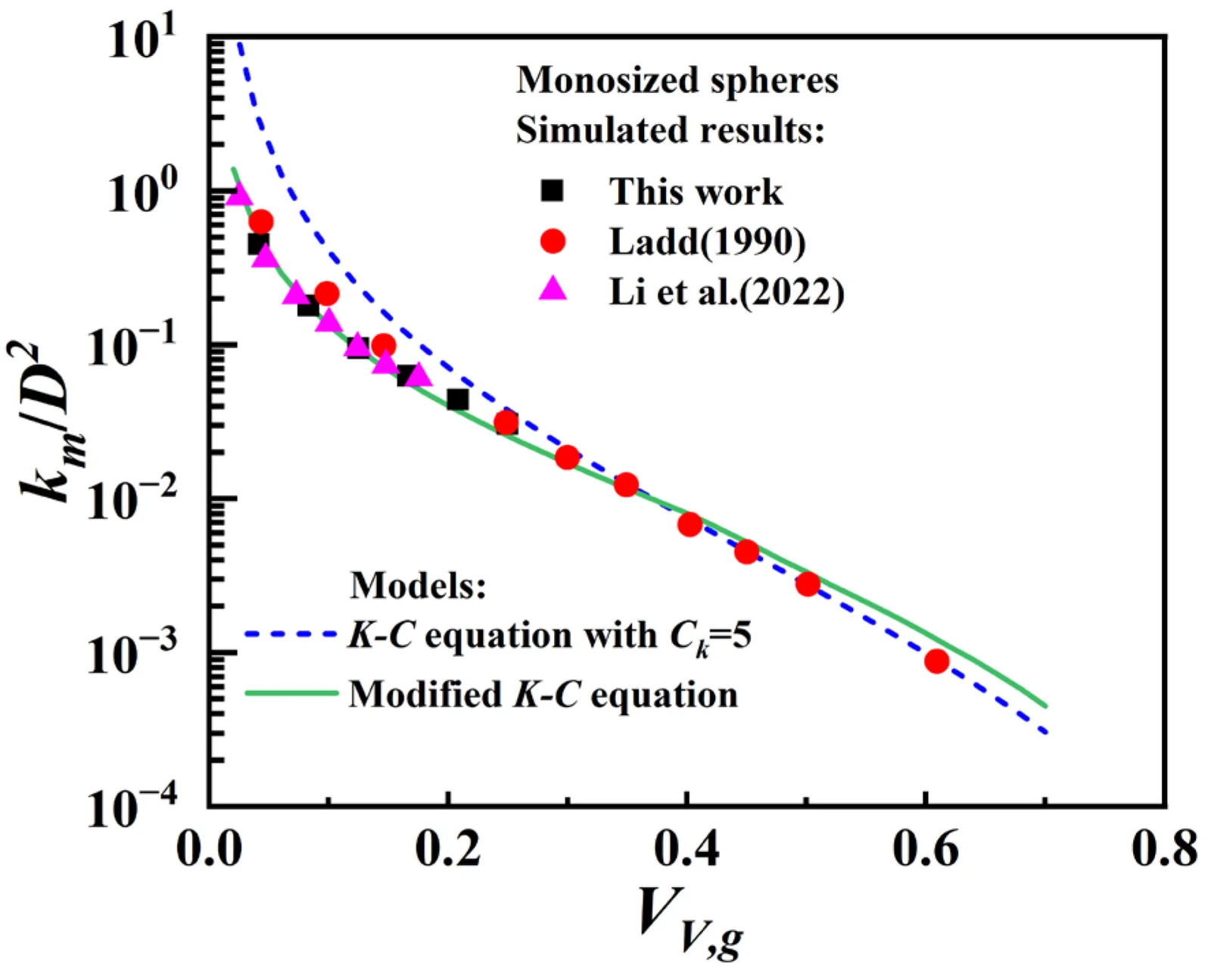
Comparison of the modified K-C equation with previous studies [32,37].

Combining the approximate formula of the shape parameter *C_f,m_* and the tortuosity *τ_p,m_* for the MPPS, a modified K-C formula is proposed to calculate the permeability *k_m_* of the MPPS, as shown in Equation (34). The permeability *k_m_* obtained from the modified K-C formula is compared with the numerical simulation data [37,38] obtained from the existing study in Figure 11, and it can be seen that the results obtained from the modified K-C formula are in agreement with the existing numerical simulation data. In addition, the modified K-C formula improves the reliability of permeability *k_m_* predictions for low-volume fraction particle packing systems compared to the regular K-C formula with *C_k_* = 5. It should be noted that all the external model validation presented in Figure 10 and Figure 11 relies solely on datasets of monodisperse spherical particles from the published literature.

(34) $$k_{m}=\frac{{\left(1-V_{V,g}\right)}^{3}}{C_{f,m}{\left(1-V_{V,g}+\frac{6V_{V,g}B_{1}P_{ave}S_{agg1}}{\pi }\right)}^{2}S_{V,g}^{2}V_{V,g}^{2}}$$

In order to evaluate how poly-sized particles affect the K-C constant *C_k,p_* for PPPS, Vidal et al. [38] presents an expression for the K-C formula constants *C_k,p_* for PPPS and the K-C constants *C_k,m_* for the MPPS, as shown in Equation (35). Considering that the particle size distributions derived above have no influence on tortuosity, so by substituting Equation (4), that is *C_k_* = *C_f_τ*^2^ to Equation (35), the relationship of *C_f,m_* and *C_f,p_* can be simplified into the ratio relationship as displayed in Equation (36). Substituting the shape parameter *C_f,m_* obtained from Equation (33) into Equation (36), the shape parameter solution formula applicable to PPPS can be derived. Then, a modified K-C formula that is applied to calculate the permeability *k_p_* for PPPS is developed in this work, as indicated in Equation (37). Moreover, it should be noted that the external permeability verification based on the published literature data is restricted to monodispersed spherical particle assemblies. Therefore, the permeability predictions for ovoid particles proposed in this work are only verified using the internal LBM simulation data adopted for shape factor fitting, without independent external benchmark validation for non-spherical granular media.

(35) $$\frac{C_{k,p}}{C_{k,m}}=\frac{0.22S_{k}+4}{4}$$

(36) $$\frac{C_{k,p}}{C_{k,m}}=\frac{\tau_{p,p}^{2}C_{f,p}}{\tau_{p,m}^{2}C_{f,m}}=\frac{C_{f,p}}{C_{f,m}}=\frac{0.22S_{k}+4}{4}$$

(37) $$k_{p}=\frac{{\left(1-V_{V,g}\right)}^{3}}{C_{f,m}\frac{0.22S_{k}+4}{4}{\left(1-V_{V,g}+\frac{6V_{V,g}B_{1}P_{ave}S_{agg1}}{\pi }\right)}^{2}S_{V,g}^{2}V_{V,g}^{2}}$$

(38) $$S_{k}=\frac{\int_{D_{agg,\min}}^{D_{agg,\max}}{\left(D-<D>\right)}^{3}f_{N}(D)dD}{{\left(\int_{D_{agg,\min}}^{D_{agg,\max}}{\left(D-<D>\right)}^{2}f_{N}(D)dD\right)}^{3/2}}$$

(39) $$<D>=\int_{D_{agg,\min}}^{D_{agg,\max}}Df_{N}(D)dD$$

where *S_k_* is the skewness of the particle size distribution [38], as shown in Equation (38); *f_N_*(*D*) is the number-based probability density function of the particle size distribution; *D* is the equivalent diameter; <*D*> represents the average of the equivalent diameters.

## 4. Conclusions

Based on the ovoidal particle packing system, this work investigates the influence of the particle shape, volume fraction and size distribution on the tortuosity and permeability of the particle packing system. In this work, the prediction formula for calculating the tortuosity is theoretically derived, and the reliability of the prediction formula is verified by comparing it with the numerical simulation results. It should be noted that the established model is only applicable to spherical, ellipsoidal and ovoid particles with random non-overlapping packing, and its performance for irregular particles remains limited. These are the primary conclusions: (1) For a given tapering parameter *T*, when *c*/*a* is less than 1.0, the tortuosity *τ_p_* falls as the aspect ratio increases; when *c*/*a* is greater than 1.0, the tortuosity *τ_p_* rises as the aspect ratio *c*/*a* increases; the minimized tortuosity is obtained when *c*/*a* = 1.0. The tortuosity *τ_p_* rises as the tapering parameter *T* increases for a particular aspect ratio *c*/*a*. (2) For the MPPS, the tortuosity *τ_p_* is the same as for poly-sized particles; that is, the tortuosity *τ_p_* is independent of the size distribution.

Based on the tortuosity prediction formula, the permeability of the particle packing system obtained from the lattice Boltzmann method, and the consequence of the shape of the particles and size distribution on the permeability characterized using a modified K-C formula, the results indicate the following: Permeability *k* increases for a certain tapering parameter *T* and subsequently falls as the aspect ratio *c*/*a* increases, and the maximum permeability is acquired with the aspect ratio of *c*/*a* = 1.0.

## Figures and Tables

**Table 1:** The value of the fitting parameters in the approximation formula.

Fitting Parameters					*R* ^2^
*a*_1_	*a*_2_	*a*_3_	*a*_4_	*a*_5_	
−6.42152	−0.00532	−0.00286	−0.04178	6.71393	0.97382

## Data Availability

The original contributions presented in this study are included in the article. Further inquiries can be directed to the corresponding author.
